# Supplementary material for: Advancing Precision Oncology Through Modeling of Longitudinal and Multimodal Data
Source: ArXiv. 2025 Jul 3:arXiv:2502.07836v3. Originally published 2025 Feb 11. Preprint. [Version 3] (PMC11844620)
Supplement: 1 [file NIHPP2502.07836V3-supplement-1.pdf]

SUPPLEMENTAL TABLE I

A SUMMARY OF PUBLICLY AVAILABLE LONGITUDINAL DATASETS IN CANCER.

Datasets[a]	Cancer Type	Study Type	Modalities			
			Clinical	Molecular[b]	Imaging[c]	Histology
NLST [1]	Lung cancer	Clinical trial: Screening	Demographics, medical/smoking/family history, diagnosis, treatment, survival	Biospecimens: blood, sputum, urine, tissue	Low Dose CT, Chest X-ray	Whole Slide Image
ACRIN 6668 Multi-center Clinical Trial [2]	Non-small cell lung cancer	Clinical trial: Response to chemoradiotherapy	Demographics, lab test, diagnosis, treatment, survival	-	FDG-PET, CT	-
PLCO Cancer Screening Trial [3]	Prostate, lung, colorectal, and ovarian cancer	Clinical trial: Screening	Demographics, medical/smoking/family history, medical history, surveys, lab test, diagnosis, treatment, survival	Biospecimens: blood, tissue<br>Biospecimen results: CA125 level, GWAS, WGS, WES, tumor sequencing, EWAS, serum metabolomics, oral microbiome	Chest X-ray	Whole Slide Image
UKLWC [4]	Ovarian cancer	Clinical trial: Screening	Demographics, medical/smoking/family history, diagnosis, treatment, survival	Biospecimens: blood<br>Biospecimen results: CA125 level	US	-
IMpower150 [5]	Non-small cell lung cancer	Clinical trial: Response to drugs	Demographics, smoking history, lab test, radiology report, survival	ctDNA data	-	-
The Cancer Moonshot Biobank [6]	Cancer: colon, lung, prostate, breast, ovarian, melanoma, gastroesophageal, myeloma, acute myeloid leukemia	Biobank	Demographics, lab test, diagnosis, treatment, survival	Biospecimens: blood, tissue<br>Biospecimen results: SNP, CNV	CT, MRI, PET, US, Angiography, X-ray	Whole Slide Image
CSAW-CC [7]	Breast cancer	Clinical trial: Screening	Demographics, medical history, diagnosis, treatment, survival	-	X-ray Mammography	-
I-SPY TRIAL [8]	Breast cancer	Clinical trial: Response to drugs	Demographics, immunohistochemistry report, surveys	Biospecimens: blood, tissue	MRI	Core-needle biopsies
EA1141 [9]	Breast cancer	Clinical trial: Screening	Demographics, medical/family history	Mutation	MRI, DBT	-
QIN Breast [10]	Breast cancer	Clinical trial: Response to neoadjuvant chemotherapy	Treatment response	-	PET/CT, DCE-MRI	-
ReMIND [11]	Brain tumor	Surgical resection	Demographics, medical history, diagnosis	Mutation	MRI, US	-
PEDSnet [12]	Brain tumor, leukemia, lymphoma	Biobank	Demographics, lab test, treatment, medication, diagnosis, outpatient/inpatient/Emergency visits	-	-	-
UK Biobank [13]	All cancer types	Biobank	Demographics, lab tests, surveys, activity monitoring, primary care data, inpatient data	Biospecimens: blood, urine, saliva<br>Biospecimen results: WGS, WES, Genotype data	MRI	-
The All of Us [14]	All cancer types	Biobank	Demographics, vital sign, lab test, medical procedure, diagnosis, surveys, digital health	Biospecimens: blood, saliva, urine<br>Biospecimen results: WGS	-	-
MIMIC [15]	All cancer types	Intensive Care Unit	Demographics, vital sign, lab test, medical procedure, medication, clinical notes	-	Chest X-ray	-
eICU [16]	All cancer types	Intensive Care Unit	Demographics, vital sign, lab test, medical procedure, medication, clinical notes	-	-	-

This table provides a subset of publicly available (or available upon request) longitudinal datasets in cancer research, with longitudinal data elements highlighted in red. These datasets typically encompass cancer screening, cancer treatment response, and large-scale biobanks. As some datasets are only accessible upon request, whether a data element is longitudinal was determined based on the available documentation.

[a]NLST = National Lung Screening Trial, PLCO = The Prostate, Lung, Colorectal and Ovarian, UKLWC = United Kingdom Collaborative Trial of Ovarian Cancer Screening Longitudinal Women's Cohort, CSAW-CC = Abbreviated Breast MRI and Digital Tomosynthesis Mammography in Screening Women With Dense Breasts, I-SPY = Investigation of Serial Studies to Predict Your Therapeutic Response with Imaging and moLecular Analysis, ReMIND = The Brain Resection Multimodal Imaging Database, PEDSnet = A National Pediatric Learning Health System, EA1141 = Abbreviated Breast MRI and Digital Tomosynthesis Mammography in Screening Women With Dense Breasts, QIN = Quantitative Imaging Network, MIMIC = The Medical Information Mart for Intensive Care. [b]GWAS = Genome-Wide Association Studies, WGS = Whole Genome Sequencing, WES = Whole exome sequencing, EWAS = epigenome-wide association, ctDNA = Circulating tumor DNA, SNP = Single Nucleotide Polymorphism, CNV = Copy Number Variation. [c]CT = Computed Tomography, FDG = Fludeoxyglucose, PET = Positron Emission Tomography, MRI = Magnetic Resonance Imaging, US = Ultrasound, DCE = Dynamic Contrast-enhanced, DBT = Digital Breast Tomosynthesis.

SUPPLEMENTAL TABLE II
FORMULAS IN LONGITUDINAL MODELING TECHNIQUES.

Method	Equations
	**Statistical Model**
**Mixed Effect Model**	The general form of a linear mixed effects model is the following:<br>$Y_{ij} = \beta_{0i} + \beta_{1i}t_j + \varepsilon_{ij}$<br>$\beta_{0i} = \beta_0 + u_{0i}$<br>$\beta_{1i} = \beta_1 + u_{1i}$,<br>where Y_{ij} is an outcome variable observed on individual i at times t_j, β_{0i} and β_{1i} are individual-specific coefficients with both fixed (β_0, β_1) and random components (u_{0i}, u_{1i}), and ε_{ij} is the residual error [17].
**Parametric Empirical Bayes**	In the context of cancer screening, the parametric empirical bayes Z score is calculated as:<br>$Z = \frac{Y - [\mu \times (1-\beta_n) + \bar{X}_i \times \beta_n]}{\sqrt{V(1-\beta_1\beta_n)}} \geq T,$<br>where Y is the current biomarker value, $\bar{X}_i$ is the mean of previous biomarker values for the individual, μ is the population mean of biomarker values, V is the population variance of biomarker values, β_1 is the intraclass correlation coefficient within individuals over time, and β_n is a weighted ICC depending on how many past values n are available [18]. The screening result is considered positive whenever Z is greater than a certain threshold T, which can be chosen based on the desired specificity.
**Dynamic Bayesian Network**	The state of each variable at time t is conditionally dependent on a subset of variables from the current and previous time step.<br>$P(X_t \mid X_{t-1}) = \prod_i P(X_t^{(i)} \mid Pa(X_t^{(i)})),$<br>where X_t is the set of all state variables at timepoint t, X_{t-1} is the set of all state variables at the previous timepoint, $X_t^{(i)}$ is the i-th variable at timepoint t, and $Pa(X_t^{(i)})$ indicates the parents of $X_t^{(i)}$, typically including variables from current and previous timepoint.
**Hidden Markov Model**	The probability of being in state j after all observations equals the total of all potential outcome paths:<br>$p_t(j) = \sum_{i=1}^{N} p_{t-1}(i) T_{ij} E_j(o_t),$<br>where $p_{t-1}(i)$ is the probability from the previous timestep in state i, T_{ij} is the transition probability from state i to j, $E_j(o_t)$ is the emission probability of having the observation at time t given in state j.
	**Deep Learning**
**Recurrent Neural Network**	At each time step t, the network takes the input x_t and the prior hidden state h_{t-1} to compute the new hidden state h_t:<br>$h_t = W_1 x_t + W_2 h_{t-1},$<br>where W_1 and W_2 represent weight matrices.
**Transformer**	Attention scores are calculated to assess how one timestamp x_t attends to all other timestamps. They are computed using query (Q) and keys (K), which are linear projections of the time-series inputs X with weights W_Q and W_K. The attention scores are obtained by computing the dot product of Q and K, where the result is scaled by the dimensionality of keys d_k and then passed through the Softmax function for normalization. The final output is derived by taking the dot product of attention scores and value matrix V, which is the linear projection of X with weights W_V [19].<br>$Q = W_Q X; K = W_K X; V = W_V X$<br>$Attention(Q, K, V) = softmax\left(\frac{QK^T}{\sqrt{d_k}}\right)V$
**Self-supervised Learning**	Self-supervised learning typically employs deep learning architectures that utilize various loss functions without using labels. Here are some example loss functions that are generally used.<br><br>Mean Square Error (MSE) loss can be used to measure the difference between the true time difference (x) and the predicted time difference ($\hat{x}$). Also, it can be used in reconstruction to minimize the difference between input (x) and reconstructed output ($\hat{x}$).<br>$L_{MSE} = \lVert x - \hat{x} \rVert^2$<br><br>Information Noise-Contrastive Estimation loss [20] can be used to pull paired embedding, z_i and z_j, closer (datapoints from different timepoints of the same individual) and push the unpaired embedding, z_i and z_n, apart. Cosine similarity is used to compute the distance between each pair of data. τ is a temperature parameter to control the softness of the probability distribution.<br>$L_{InfoNCE} = -\log \frac{\exp(sim(z_i, z_j)/\tau)}{\sum_{n=i}^{N} \exp(sim(z_i, z_n)/\tau)}$
**Reinforcement Learning**	Bellman equation is the fundamental concept in reinforcement learning. It is a recursive function which presents the value of being in the current state s, $V(s)$, using immediate reward (R) when action a was taken and the value of the subsequent state $V(s')$.<br>$V(s) = \max_a (R(s, a) + \gamma V(s'))$

References

[1] "Reduced Lung-Cancer Mortality with Low-Dose Computed Tomographic Screening," *N. Engl. J. Med.*, vol. 365, no. 5, pp. 395–409, Aug. 2011.

[2] M. Machtay *et al.*, "Prediction of Survival by [18F]Fluorodeoxyglucose Positron Emission Tomography in Patients With Locally Advanced Non–Small-Cell Lung Cancer Undergoing Definitive Chemoradiation Therapy: Results of the ACRIN 6668/RTOG 0235 Trial," *J. Clin. Oncol.*, vol. 31, no. 30, pp. 3823–3830, Oct. 2013.

[3] P. C. Prorok *et al.*, "Design of the prostate, lung, colorectal and ovarian (PLCO) cancer screening trial," *Control. Clin. Trials*, vol. 21, no. 6, Supplement 1, pp. 273S-309S, Dec. 2000.

[4] I. J. Jacobs *et al.*, "Ovarian cancer screening and mortality in the UK Collaborative Trial of Ovarian Cancer Screening (UKCTOCS): a randomised controlled trial," *Lancet Lond. Engl.*, vol. 387, no. 10022, pp. 945–956, Mar. 2016.

[5] M. A. Socinski *et al.*, "Atezolizumab for First-Line Treatment of Metastatic Nonsquamous NSCLC," *N. Engl. J. Med.*, vol. 378, no. 24, pp. 2288–2301, Jun. 2018.

[6] D. S. Singer, "A new phase of the Cancer Moonshot to end cancer as we know it," *Nat. Med.*, vol. 28, no. 7, pp. 1345–1347, Jul. 2022.

[7] K. Dembrower *et al.*, "A Multi-million Mammography Image Dataset and Population-Based Screening Cohort for the Training and Evaluation of Deep Neural Networks—the Cohort of Screen-Aged Women (CSAW)," *J. Digit. Imaging*, vol. 33, no. 2, pp. 408–413, Apr. 2020.

[8] A. Barker *et al.*, "I-SPY 2: An Adaptive Breast Cancer Trial Design in the Setting of Neoadjuvant Chemotherapy," *Clin. Pharmacol. Ther.*, vol. 86, no. 1, pp. 97–100, 2009.

[9] C. E. Comstock *et al.*, "Comparison of Abbreviated Breast MRI vs Digital Breast Tomosynthesis for Breast Cancer Detection Among Women with Dense Breasts Undergoing Screening," *JAMA*, vol. 323, no. 8, pp. 746–756, Feb. 2020.

[10] L. P. Clarke *et al.*, "Quantitative Imaging for Evaluation of Response to Cancer Therapy," *Transl. Oncol.*, vol. 2, no. 4, p. 195, Dec. 2009.

[11] P. Juvekar *et al.*, "ReMIND: The Brain Resection Multimodal Imaging Database," Apr. 08, 2024, *medRxiv*.

[12] C. B. Forrest *et al.*, "PEDSnet: a National Pediatric Learning Health System," *J. Am. Med. Inform. Assoc.*, vol. 21, no. 4, pp. 602–606, Jul. 2014.

[13] C. Bycroft *et al.*, "The UK Biobank resource with deep phenotyping and genomic data," *Nature*, vol. 562, no. 7726, pp. 203–209, Oct. 2018.

[14] The All of Us Research Program Investigators, "The 'All of Us' Research Program," *N. Engl. J. Med.*, vol. 381, no. 7, pp. 668–676, Aug. 2019.

[15] A. E. W. Johnson *et al.*, "MIMIC-III, a freely accessible critical care database," *Sci. Data*, vol. 3, no. 1, p. 160035, May 2016.

[16] T. J. Pollard *et al.*, "The eICU Collaborative Research Database, a freely available multi-center database for critical care research," *Sci. Data*, vol. 5, no. 1, p. 180178, Sep. 2018.

[17] M. Herle *et al.*, "Identifying typical trajectories in longitudinal data: modelling strategies and interpretations," *Eur. J. Epidemiol.*, vol. 35, no. 3, pp. 205–222, Mar. 2020.

[18] C. W. Drescher *et al.*, "Longitudinal Screening Algorithm That Incorporates Change Over Time in CA125 Levels Identifies Ovarian Cancer Earlier Than a Single-Threshold Rule," *J. Clin. Oncol.*, vol. 31, no. 3, pp. 387–392, Jan. 2013.

[19] A. Vaswani *et al.*, "Attention is All you Need," in *Advances in Neural Information Processing Systems*, Curran Associates, Inc., 2017.

[20] A. van den Oord *et al.*, "Representation Learning with Contrastive Predictive Coding," Jan. 22, 2019, *arXiv*: arXiv:1807.03748.
